# Oral administration of encapsulated catechin in chitosan‐alginate nanoparticles improves cognitive function and neurodegeneration in an aluminum chloride‐induced rat model of Alzheimer's disease

**DOI:** 10.14814/phy2.16095

**Published:** 2024-07-01

**Authors:** Elnaz Mohammadbaghban, Ali Taravati, Hossein Najafzadehvarzi, Hossein Khaleghzadeh‐Ahangar, Fatemeh Tohidi

**Affiliations:** ^1^ Cellular and Molecular Biology Research Center, Health Research Institute Babol University of Medical Sciences Babol Iran; ^2^ Student Research Committee Babol University of Medical Sciences Babol Iran; ^3^ Biomedical and Microbial Advanced Technologies (BMAT) Research Center, Health Research Institute Babol University of Medical Sciences Babol Iran; ^4^ Department of Molecular and Cell Biology, Faculty of Basic Sciences University of Mazandaran Babolsar Iran; ^5^ Department of Physiology, School of Medicine Babol University of Medical Sciences Babol Iran; ^6^ Mobility Impairment Research Center, Health Research Institute Babol University of Medical Sciences Babol Iran; ^7^ Cancer Research Center, Health Research Institute Babol University of Medical Sciences Babol Iran

**Keywords:** acetylcholinesterase, Alzheimer's disease, catechin, chitosan‐alginate nanoparticles

## Abstract

The present study aimed to investigate the effect of catechin‐loaded Chitosan‐Alginate nanoparticles (NPs) on cognitive function in an aluminum chloride (AlCl_3_)‐induced rat model of Alzheimer's disease (AD). The Catechin‐loaded Chitosan‐Alginate nanocarriers were synthesized through ionotropic gelation (IG) method. Physio‐chemical characterization was conducted with the Zetasizer Nano system, the scanning electron microscope, and the Fourier transform infrared spectroscopy. The experiments were performed over 21 days on six groups of male Wistar rats. The control group, AlCl_3_ treated group, Catechin group, nanocarrier group, treatment group 1 (AlCl_3_ + Catechin), and treatment group 2 (AlCl_3_ + nanocarrier). A behavioral study was done by the Morris water maze (MWM) test. In addition, the level of oxidative indices and acetylcholine esterase (AChE) activity was determined by standard procedures at the end of the study. AlCl_3_ induced a significant increase in AChE activity, along with a significant decrease in the level of Catalase (CAT) and total antioxidant capacity (TAC) in the hippocampus. Moreover, the significant effect of AlCl_3_ was observed on the behavioral parameters of the MWM test. Both forms of Catechin markedly improved AChE activity, oxidative biomarkers, spatial memory, and learning. The present study indicated that the administration of Catechin‐loaded Chitosan‐Alginate NPs is a beneficial therapeutic option against behavioral and chemical alteration of AD in male Wistar rats.

## 1 INTRODUCTION

Dementia has become a major public health issue worldwide. In 2016, the global number of individuals living with dementia was 57.4 million, which is expected to reach 152.8 million by 2050 (Nichols et al., 2022; Zhang et al., 2021). Alzheimer's disease (AD) is the most common type of dementia, accounting for almost 60% to 70% of dementia cases worldwide (Prince et al., 2015). This neurodegenerative disease is initially characterized by progressive memory decline followed by abnormalities in behavioral and cognitive functions such as language, executive, and visuospatial impairment (AnjiReddy & Karpagam, 2017; McKhann et al., 2011; Tapeinos et al., 2017).

Three hallmark pathological features of AD include the extracellular senile plaque, tau protein hyperphosphorylation, and neurodegeneration in frontal and hippocampal cholinergic neurons (Jia et al., 2016). Cognitive memory pattern is directly related to central and peripheral cholinergic neurotransmission systems. Acetylcholine (Ach) is predominantly active in learning and nerve conduction systems due to its' potential role in communication between neurons (Ouyang et al., 2017). Experts believe that many people with AD have lower levels of Ach, highlighting perhaps another function of the mentioned neurotransmitter. A decrease in the activity of AchE may result in an increased level of acetylcholine in the brain. This rise can enhance neuron signal transmission and potentially increase stimulation of cholinergic receptors (Hamulakova et al., 2016). Despite substantial research advances, there is still no efficacious medication for AD prevention and treatment. Thus, much attention has been paid to the development of various alternative strategies to prevent and slow down the progression of AD.

In recent decades, a great deal of attention has been devoted to herbal medicines as alternative treatment options for AD (Bui & Nguyen, 2017; Jiang et al., 2017; Peng et al., 2021). Green tea is one of the most popular beverages and medicinal plants worldwide. Catechins and their derivatives, specifically (−)‐epigallocatechin gallate (EGCG), are bioactive compounds in green tea that are responsible for the beneficial role of the plant in health. A growing number of preclinical studies have supported the favorable effect of green tea catechins on the prevention and treatment of dementia and AD (Youn et al., 2022). However, there are some indications of the limited effectiveness of green tea on dementia and cognitive function in human subjects (Fischer et al., 2018; Ide et al., 2015; Shen et al., 2015; Xu et al., 2018). Limited stability, bioavailability and oral absorption of green tea catechins can explain the inconsistent results between preclinical and clinical studies (Lee et al., 2002; Li et al., 2000; Zhu et al., 2000).

Several strategies have been proposed to increase the bioavailability of green tea catechins, of which nano‐encapsulation has been known as a novel and promising method. Various kinds of food biopolymers such as lipids, proteins, and polysaccharides have been used as nanocarriers for catechins (Dag & Oztop, 2017; Dube et al., 2011; Shi et al., 2018). Chitosan is an alkaline natural polysaccharide that has been studied extensively for the nano‐encapsulation of catechins. The results of previous studies have suggested that encapsulation of catechins in chitosan nanoparticles (NPs) improves their bioavailability by enhancing their intestinal absorption, stability, sustained release, and plasma concentration (Dube et al., 2011; Dube, Ng, et al., 2010; Dube, Nicolazzo, & Larson, 2010; Zou et al., 2015). Furthermore, the therapeutic efficacy of encapsulated EGCG in chitosan NPs has been shown by some studies (Hong et al., 2014; Khan et al., 2014). The results of a study by Khan et al indicated that oral administration of chitosan nano‐EGCG inhibits the growth of prostate cancer cells in a xenograft model of athymic nude mice (Khan et al., 2014). Additionally, the encapsulation of EGCG in chitosan and polyaspartic acid improved the efficacy of EGCG against rabbit atherosclerosis (Hong et al., 2014). However, to the best of our knowledge, no previous study has investigated the influence of catechin‐loaded chitosan‐alginate NPs on cognitive function and dementia. Thus, the present study aimed to investigate the effect of catechin‐loaded chitosan‐alginate NPs on cognitive function and neurodegeneration in an aluminum chloride‐induced rat model of AD.

## 2 MATERIALS AND METHODS

### 2.1 Materials

Catechin hydrate (C1251, purity 98%), medium molecular weight chitosan with a deacetylation degree of 85%, 5,5′‐dithiobis‐2‐nitrobenzoesaure (DNTB, CAS 69–78‐3), acetylthiocholine iodide (ATCI), tripyridyltriazine (TPTZ, T1253), Aluminum chloride (AlCl_3_, anhydrous CAS# 7446‐70‐0) and alginate powder were purchased from Sigma Aldrich company and 98% ethanol and sodium tripolyphosphate (TPP) were purchased from Merck (Germany).

### 2.2 Preparation of catechin‐loaded chitosan‐alginate‐NPs

Ionotropic gelation (IG) technique was used to prepare chitosan‐alginate NPs loaded with catechin. Briefly, 0.0064 g of chitosan powder was dissolved in 8 mL of 1% acetic acid and the pH was adjusted to 4.5. Afterward, 500 μL of catechin solution was added to the mixture. Then, 4 mL of calcium chloride solution (3.35 mg/mL) and chitosan solution (0.8 mg/mL) were added dropwise and gently to 20 mL of alginate solution (3 mg/mL) within 2 h under magnetic stirring (250 rpm) at room temperature. To obtain chitosan‐alginate gel, the dispersion was centrifuged at 13000 rpm for 15 min, freeze‐dried, and stored at − 20°C.

#### 2.2.1 Physio‐chemical characterizations of catechin‐loaded NPs

##### Particles size & zeta potential measurements

To determine the zeta potential of NPs, the dynamic light scattering (DLS) method was used. To study the property of samples, it was dispersed in distilled water and then examined using the Zetasizer Nanosystem (Malvern Instrument, UK).

##### Drug entrapment efficiency and loading capacity

The amount of catechin loaded on chitosan‐alginate nanoparticles was examined through the Folin–Ciocalteu (F‐C) method. Different solutions containing various concentrations of catechin were prepared, and the concentration of catechin in the supernatant and the initial solution was measured by an ultraviolet‐spectrophotometer at the wave length of 765 nm. The standard curves were utilized to estimate catechin amounts. Encapsulation efficiency (EE%) and loading capacity (LC%) were o calculated based on the following equations:

$$\mathrm{EE}\;\left(\%\right)=\frac{\text{Total drug}-\text{Free drug}}{\text{Total drug}}\times 100.$$

$$\mathrm{LC}\;\left(\%\right)=\frac{\text{Total drug}-\text{Free drug}}{\mathrm{NPs}\;\text{Weight}}\times 100$$

#### 2.2.2 Particle's morphology

Morphology of chitosan‐alginate NPs was investigated by a field emission scanning electron microscope (FESEM; TESCAN Mira3). The lyophilized NPs were placed on the aluminum tape. Then, the samples were coated by a layer of gold under a vacuum.

#### 2.2.3 Fourier transform infrared spectroscopy

To clarify the structure of chitosan‐alginate NPs and catechin, Fourier transform infrared (FT‐IR) spectroscopy has been used. Concisely, the dry powder was mixed with potassium bromide (KBr) (1:10) and compressed into pellets. Finally, FT‐IR spectra in the 400–4000 cm^−**1**^ range were measured using a spectrophotometer (FT‐IR Alpha, Bruker, USA).

#### 2.2.4 Analyses of in vitro drug release

The in vitro release of catechin from chitosan‐alginate NPs was surveyed at different ratios of drug‐to‐polymer. After determining the chitosan‐alginate gel weight, catechin release was analyzed in three buffers: KCl‐HCl (pH = 1), Acetate buffer (pH = 5), and Tris–HCl buffer (pH = 7). Samples were collected at regular intervals for 4 h. The fresh dissolution medium was replaced in order to maintain the volume of the buffer. The amount of drug released was measured through the F‐C method as described previously.

#### 2.2.5 Pharmacodynamic studies

##### Animal subjects and experimental procedure

**FIGURE 1:**
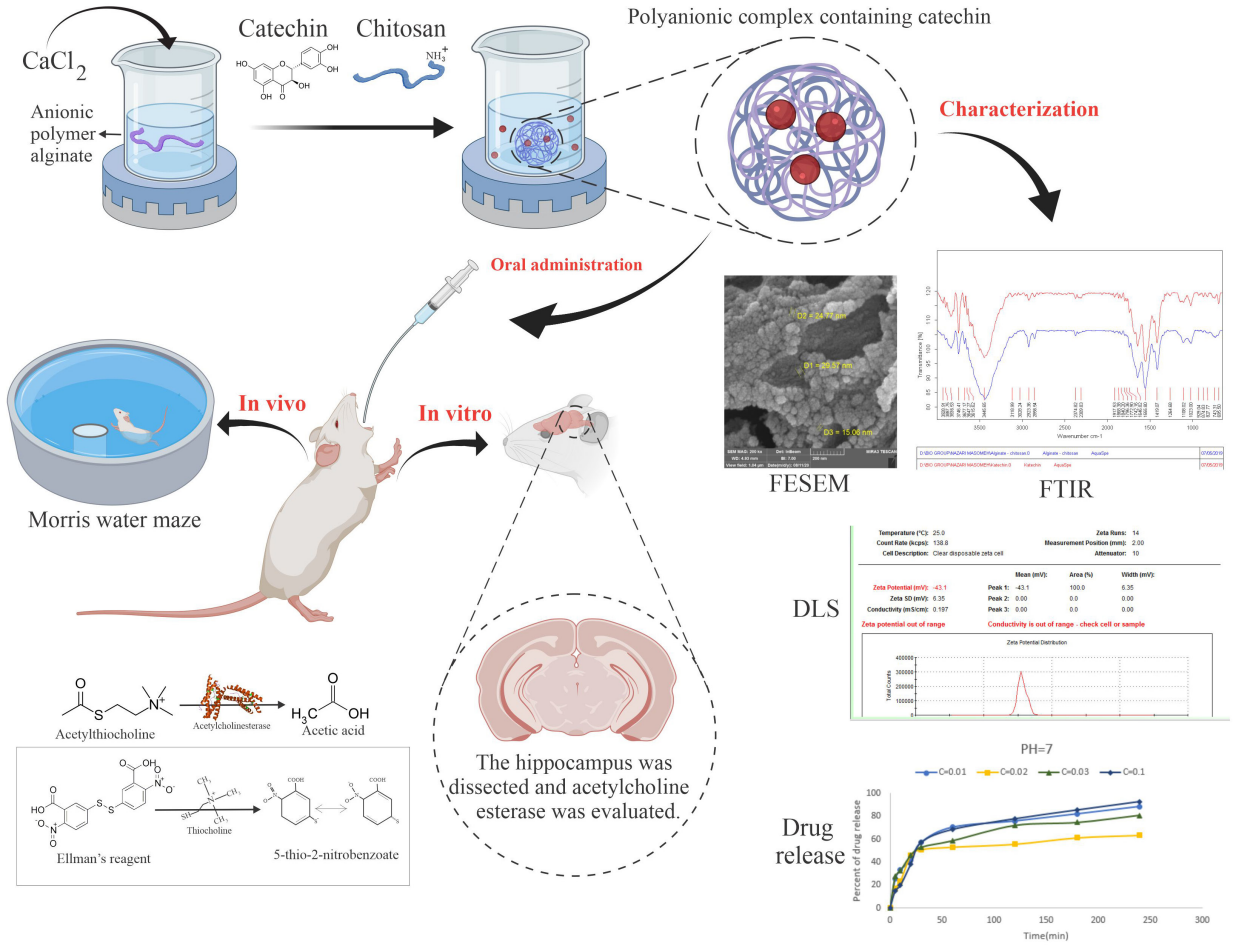
Schematic diagram of the experiment. Evaluation of the protective effect of catechin nanoparticles in the induced rat model of dementia.

All animals used in this study were obtained from the center care and breeding of laboratory animals at Babol University of Medical Sciences, Iran. The animals were 200–250 g and adult (8–12 weeks) male Wistar rats. During the experimental period, all rats were kept in propylene cages, with proper ventilation and suitable condition regarding the temperature (23 ± 2°C), humidity (60 ± 10%), light (12/12 h light/dark cycle), and free access to water and standard food pellets. All efforts were made to minimize the number and harassment of the animals. The protocol of the study was approved by the Ethics Committee for Research on Animals (ECRA), Babol University of Medical Sciences, Babol, Iran (IR.MUBABOL.HRI.REC1398.012) and performed following the National Institute of Health Guide for the Care and Use of Laboratory Animals (National Research Council, Division on Earth, Life Studies, Institute for Laboratory Animal Research, Committee for the Update of the Guide for the Care, Use of Laboratory Animals, 2010). The experiments were performed over a period of 21 days and the bio‐distribution of catechin‐loaded chitosan‐alginate NPs was evaluated on six groups of rats (*n* = 12 rats in each group) as follows: (1) the control group received 0.2 mL of 0.9% normal saline intraperitoneally (IP); (2) the aluminum chloride group received 100 mg/kg AlCl_3_ IP (Lakshmi et al., 2015); (3) the catechin group was gavaged with 0.2 mL of the prepared catechin solution (50 mg/kg) (Rai et al., 2019); (4) the nanocarrier group was gavaged with 0.2 mL of prepared solution of catechin‐loaded chitosan‐alginate NPs (10 mg/kg); (5) the treatment group 1 (AlCl_3_ + catechin) received 0.2 mL of AlCl_3_ solution (100 mg/kg) IP and was gavaged with 0.2 mL of catechin solution (50 mg/kg) after 1 h; (6) the treatment group 2 (AlCl_3_ + nanocarrier) received 0.2 mL of prepared AlCl_3_ solution (100 mg/kg) IP and gavaged with 0.2 mL of the prepared nanocarrier solution (10 mg/kg). Finally, rats were divided into two groups randomly; the first group underwent the Morris water maze (MWM) test to investigate their spatial learning and memory. In the second group, the hippocampal tissue was dissected and used to measure acetylcholinesterase (AChE) activity and the levels of other biochemical parameters (Figure 1).

##### Behavioral study

MWM is a black circular pool (136 cm in diameter and 55 cm in height) filled with water to a depth of 35 cm. A hidden platform in the center of the target quadrant was located beneath the water surface. To collect and analyze data from the rats' swimming route, we used a video tracking system (EthoVision XT7 software, Netherlands). Twenty‐four hours before training, the animals were allowed to swim in the pool for 60 s without the platform to adapt to the environment (Mohammadpour et al., 2015). A series of learning trials were then conducted over four consecutive days (four trials per rat per day). Then, rats were placed in the pool at four designated starting points and allowed to swim toward the hidden platform. Each rat was tested four times (one trial per quadrant). After each experiment, the rats were kept in a holding cage for 30 s so that they could rest before starting the following experiment. A maximum of 60 s was given to each rat in each trial to find the hidden platform; if the animal failed to discover the platform during this time, it was guided manually. We assessed MWM task learning by measuring the time to reach the platform and the swimming distance. After finding the platform, rats were allowed to remain there for a few seconds to assess its position. On the probe day, the hidden platform was removed, and the rats were placed in front of the target area, facing the wall. The Rats were allowed to swim freely for 60 s and the time spent in the target quadrant was recorded in order to assess their spatial memory (Ghobadian et al., 2018; Ghobadian et al., 2020; Ramin et al., 2011). All steps were performed under a blind procedure to ensure unbiased evaluation of animal assays.

#### 2.2.6 Biochemical assays

##### Preparation of samples

On the 22nd day, animals were sacrificed with carbon dioxide and were decapitated by guillotine. Thereafter, the brain was taken out, and the hippocampal tissue was removed. Each hippocampal tissue was weighted separately and homogenized with 10% saline phosphate buffer (pH = 7.4) using a homogenizer (Speedmill plus, Analytik Jena AG, Germany) for 4 min. For further testing, tissue homogenate samples were centrifuged at 12000 rpm for 10 min at 4°C. Supernatants were collected and stored at −80°C. Enzyme degradation was prevented by keeping the whole process on ice.

##### Acetylcholinesterase enzyme assay

The Ellman method determined an enzyme activity of AChE by following a protocol developed by S. Padilla. The principle of reaction was enzymatic degradation of acetylthiocholine iodide to thiocholine and its reaction with DTNB (Padilla et al., 1999). DTNB solution and 0.1 M phosphate buffer (pH = 8) were mixed in a 1:30 ratio to prepare the buffer/DTNB solution. Then, 20 μL of sample containing buffer/DTNB working solution was poured into each well of the plate and the substrate (Acetylthiocholine iodide) was added after 10 min. Finally, yellow color intensity of the product, which indicates the AChE activity level, was measured by the spectrophotometer at the wave length of 412 nm.

##### Catalase enzyme assay

The activity of catalase (CAT) activity was measured by a single‐step, simple and sensitive method suitable for the kinetic process based on the decomposition of H_2_O_2_, without a need for adding other substrates (Aebi, 1984). The assay was performed in a microplate by adding 300 μL of a reagent (prepared from 0.1 M phosphate buffer (pH = 6–6.5) and 24 μL of H_2_O_2_) to 5 μL of hippocampal tissue supernatant samples. The optical density of each sample was kinetically read at the wavelength of 240 nm for 2 min.

##### Total antioxidant capacity assessment

Ferric Reducing ability of plasma (FRAP) assay is a simple measure of total antioxidant capacity (TAC). This method measures the capacity of antioxidants to reduce ferric‐tripyridyltriazine (TPTZ) to ferrous‐TPTZ which is detectable at the wavelength of 593 nm (Szőllősi & Varga, 2002). The working solution was prepared by adding 50 mL acetate buffer to 5 mL TPTZ and 5 mL ferric chloride. Then, 1.5 mL working solution was combined with 50 mL standard sample and incubated at 37°C for 5 min. After reading the optical density at the wave length of 593 nm, the standard curve was plotted. To estimate TAC, 20 μL hippocampal homogenate supernatant was mixed with 1.5 mL working solution, incubated at 37°C, and the optical density was then measured at the wave length of 593 nm.

##### Statistical analysis

Results are presented as mean ± standard deviation (SD) and mean ± standard error (SE) for quantitative and categorical data, respectively. The comparison between various treatment groups was done by the one‐way analysis of variance (ANOVA). Furthermore, Tukey's post hoc test was performed at a statistical significance level of *p* < 0.05 for pairwise comparisons between different groups.

## 3 RESULTS

### 3.1 Physio‐chemical characterizations of catechin‐loaded NPs

**FIGURE 2:**
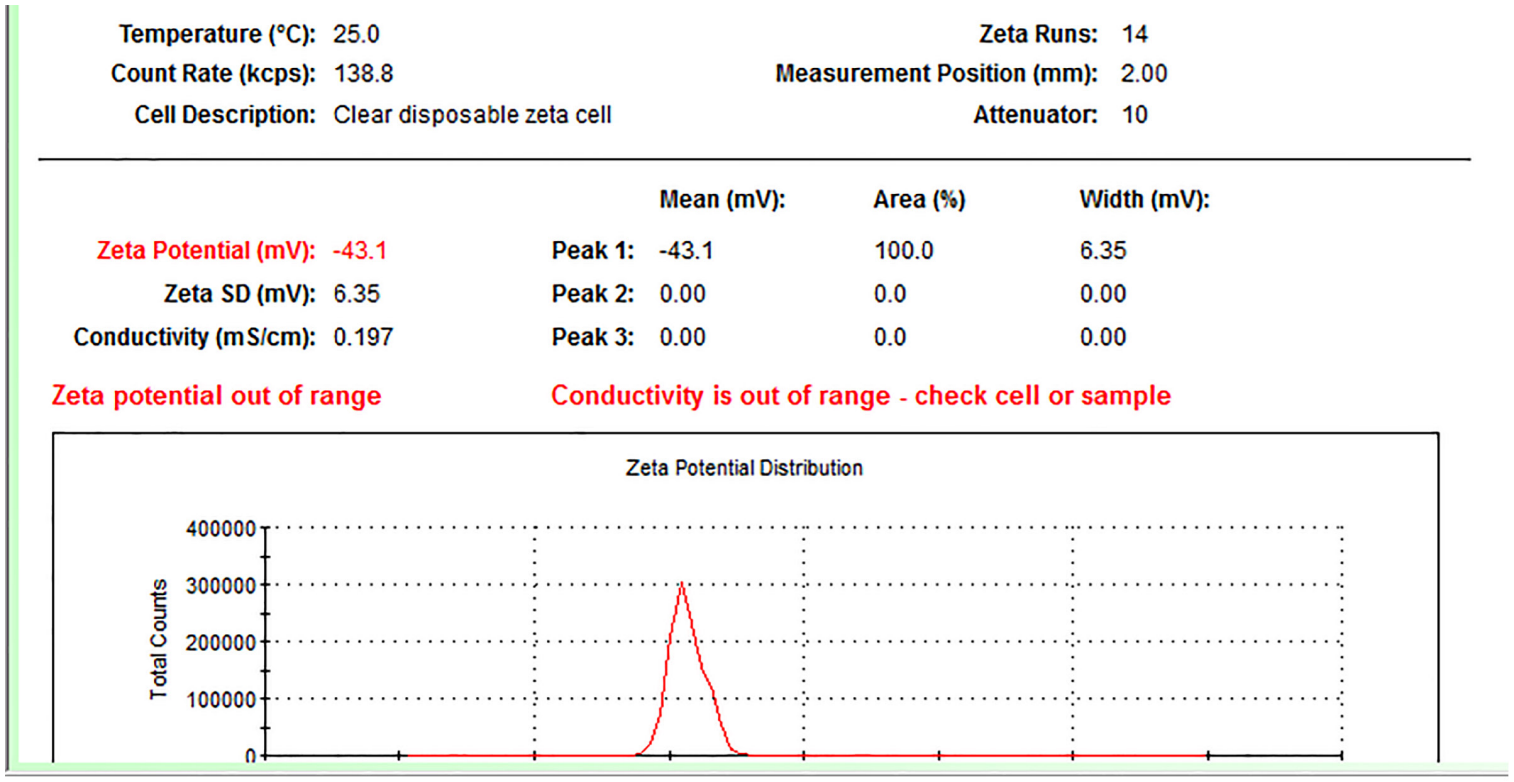
Characterization of nanoparticles. Zeta potential of chitosan‐alginate nanoparticles obtained −43.1 mv by DLS method.

**FIGURE 3:**
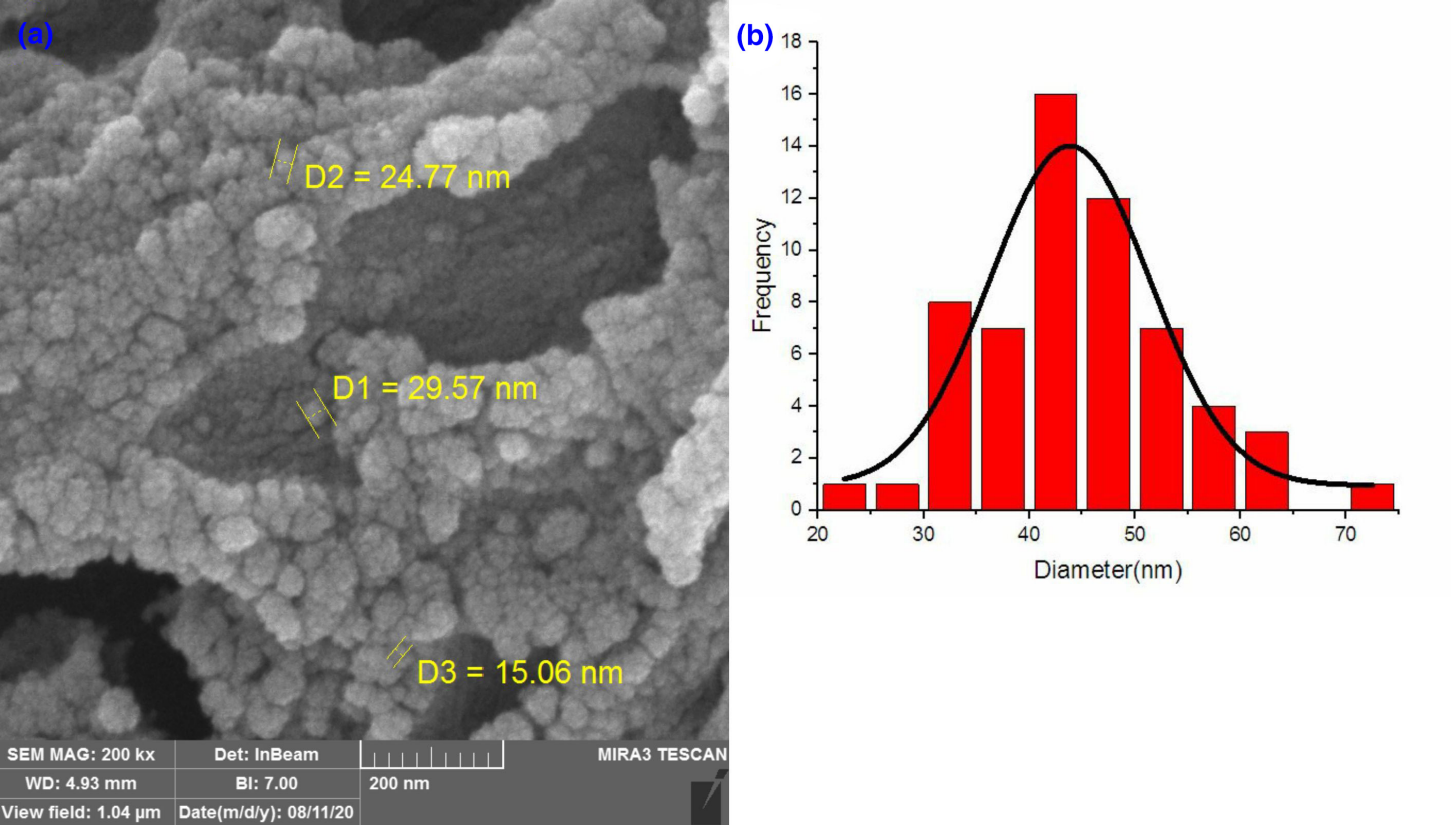
Particle's morphology. (a) Scanning electron microscopic image of chitosan‐alginate nanoparticles. Nanoparticles were spherical and well dispersed. (b) The nanoparticles distribution histogram. particle size range was between 25 and 75 nm and mostly about 45 nm.

**FIGURE 4:**
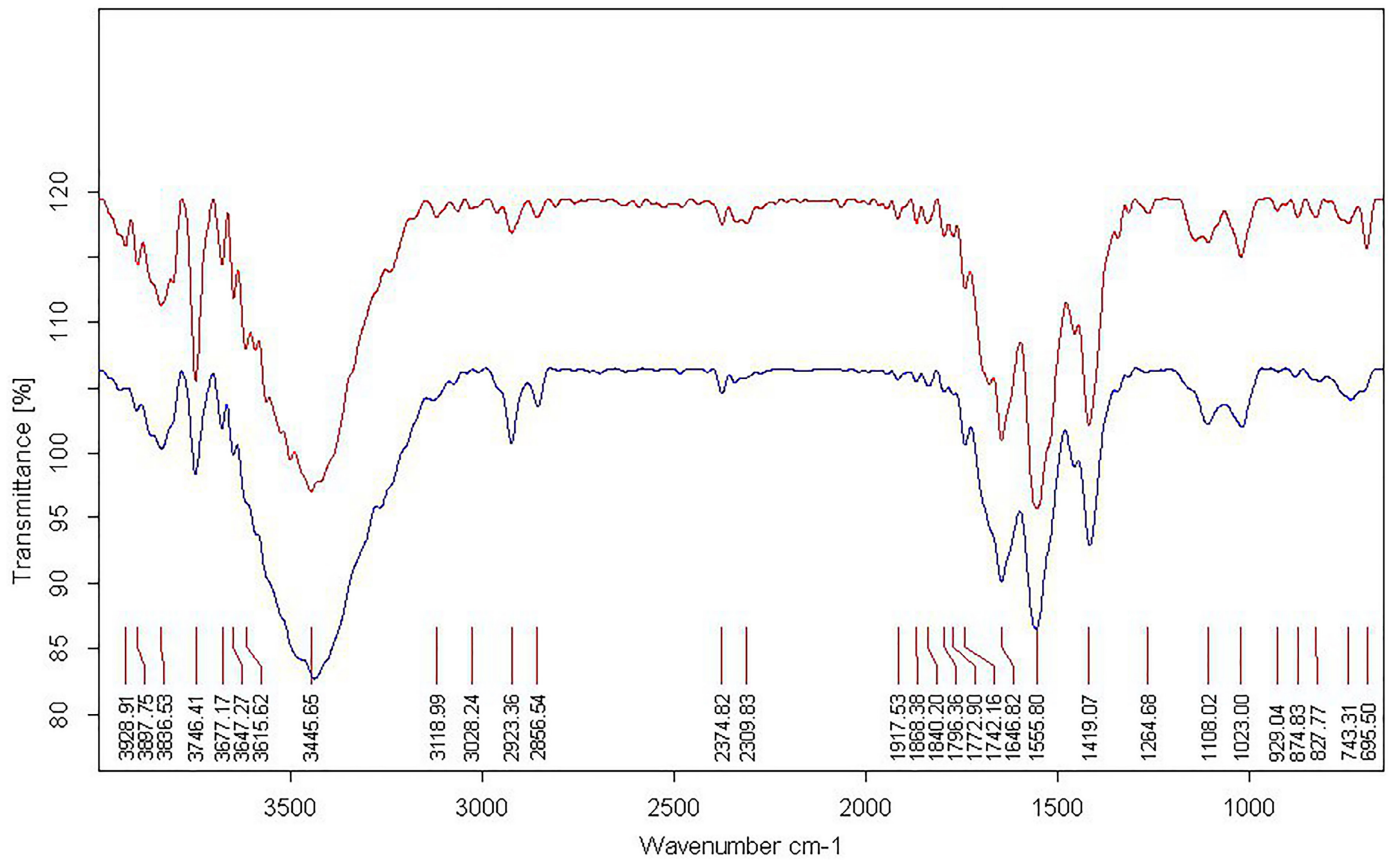
Physio‐chemical characterization of nanoparticles. FT‐IR spectra of chitosan‐alginate nanoparticles and drug catechin showed the chemical stability and formation of polyelectrolyte network by electrostatic interactions between carboxylate alginate groups and ammonium chitosan groups.

The particle's surface charge and zeta potential were presented in Figure 2. Moreover, encapsulation efficiency and drug loading capacity experiments were performed in triplicate, and entrapment efficacy was calculated at 60%. As shown in Figure 3b, NPs had a spherical morphology and dispersed well. A distribution histogram of NPs showed that maximum dispersion occurred around 40–45 nm (Figure 3b). The chemical stability and functional groups of NPs were analyzed through FT‐IR spectra (Figure 4). A broad and sharp peak was presented at 3440.26 cm^−1^ which corresponds to the stretching vibration of intramolecular hydroxyl (O‐H) and amino (‐NH_2_) groups. In addition, an average peak was detected at 1417 cm^−1^ that is associated with a symmetric stretch of carboxylate (−coo‐), chitosan, and alginate. Two identified peaks at 1560 cm^−1^ and 1647 cm^−1^ can be attributed to the stretching vibration of the carbonyl (C=O) group (amide I). These findings indicated that the polyelectrolyte network is formed by electrostatic interactions between the carboxylate alginate and ammonium chitosan groups.

### 3.2 In vitro catechin release

**FIGURE 5:**
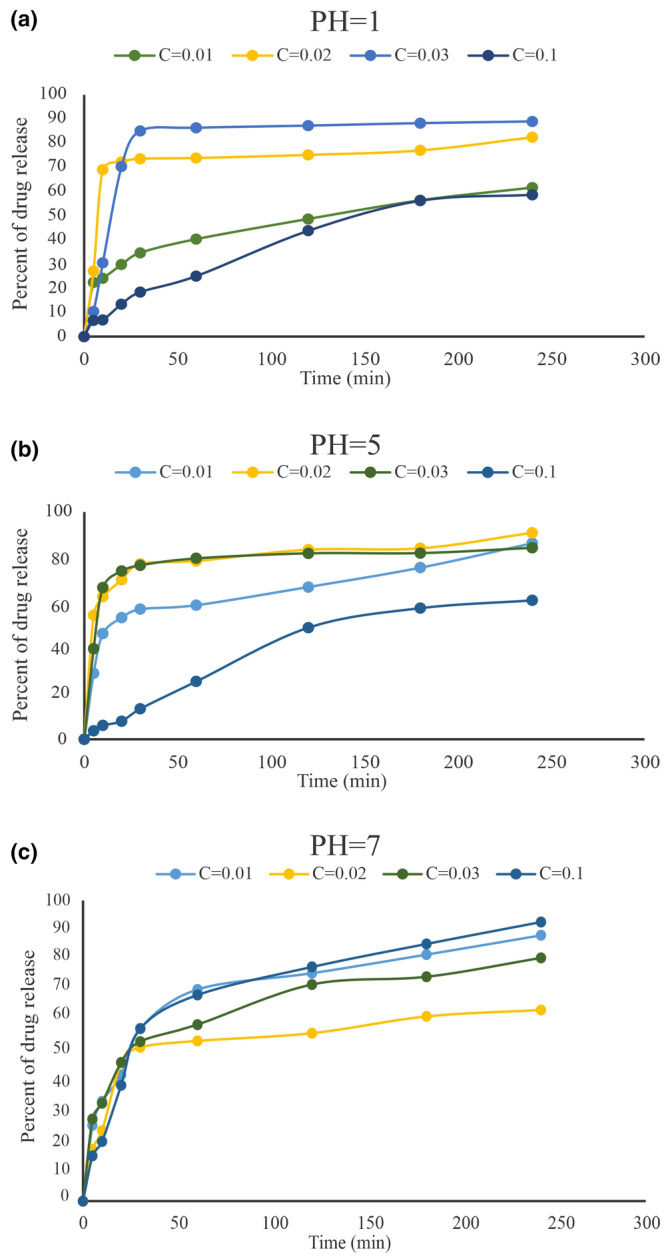
Release study. In vitro release profile of chitosan‐alginate nanoparticles in different concentration of the drug catechin and various pH.

The amount of catechin released at pH 1, pH 5 and pH 7 over a period of 5 h from NPs, is shown in Figure 5. A total of four different NPs were constructed at concentrations of 0.01, 0.02, 0.03, and 0.1 mg of catechins. At pH 1, catechin released from NPs prepared with 0.1 mg of catechin was lower than other concentration. In contrast, at pH 7 the amount of catechin released from those NPs was higher than for other concentrations. Alternatively, there was a fast release of catechin in all pH for NPs prepared with lower concentrations of catechin, 0.01, 0.02, and 0.03 mg. This may be attributed to the higher water‐uptake properties of NPs and easily release of water‐accessible catechin on the surface of NPs. Based on these finding, it is proposed that during the first hour after consuming catechin, it reaches approximately 79% (at pH = 5) and 86% (at pH = 1) based on the concentrations less than 0.1. Since our digestive tracts are acidic, this rate of release is unacceptable, and shows that a large amount of the drug is released at these concentrations. However, when the catechin concentration is raised within NPs to 0.1 only 25% of the drug is released in acidic pH during the first hour. This shows that the drug is only lost in a small amount in the stomach acidic environment. Observations carried out at pH 7 revealed that a large amount of the drug (68%) is released at this pH since the pH of the blood circulation system is also seven, which means that when the drug gets into the blood stream a high rate of it gets released (68.57%). (Figure 5). As a result, if it remains in the blood circulation for a longer period of time, the amount of drug reaching the brain increases.

### 3.3 Acetylcholinesterase enzyme activity in brain

The amount of the AchE enzyme activity in the control group, AlCl_3_ treated group, catechin group, nanocarrier group, treatment group 1 (AlCl_3_ + catechin), and treatment group 2 (AlCl_3_ + nanocarrier) were 0.78 ± 0.15, 1.44 ± 0.25, 0.86 ± 0.21, 0.81 ± 0.57, 0.90 ± 0.15 and 0.96 ± 0.27 U/mg protein, respectively.

**FIGURE 6:**
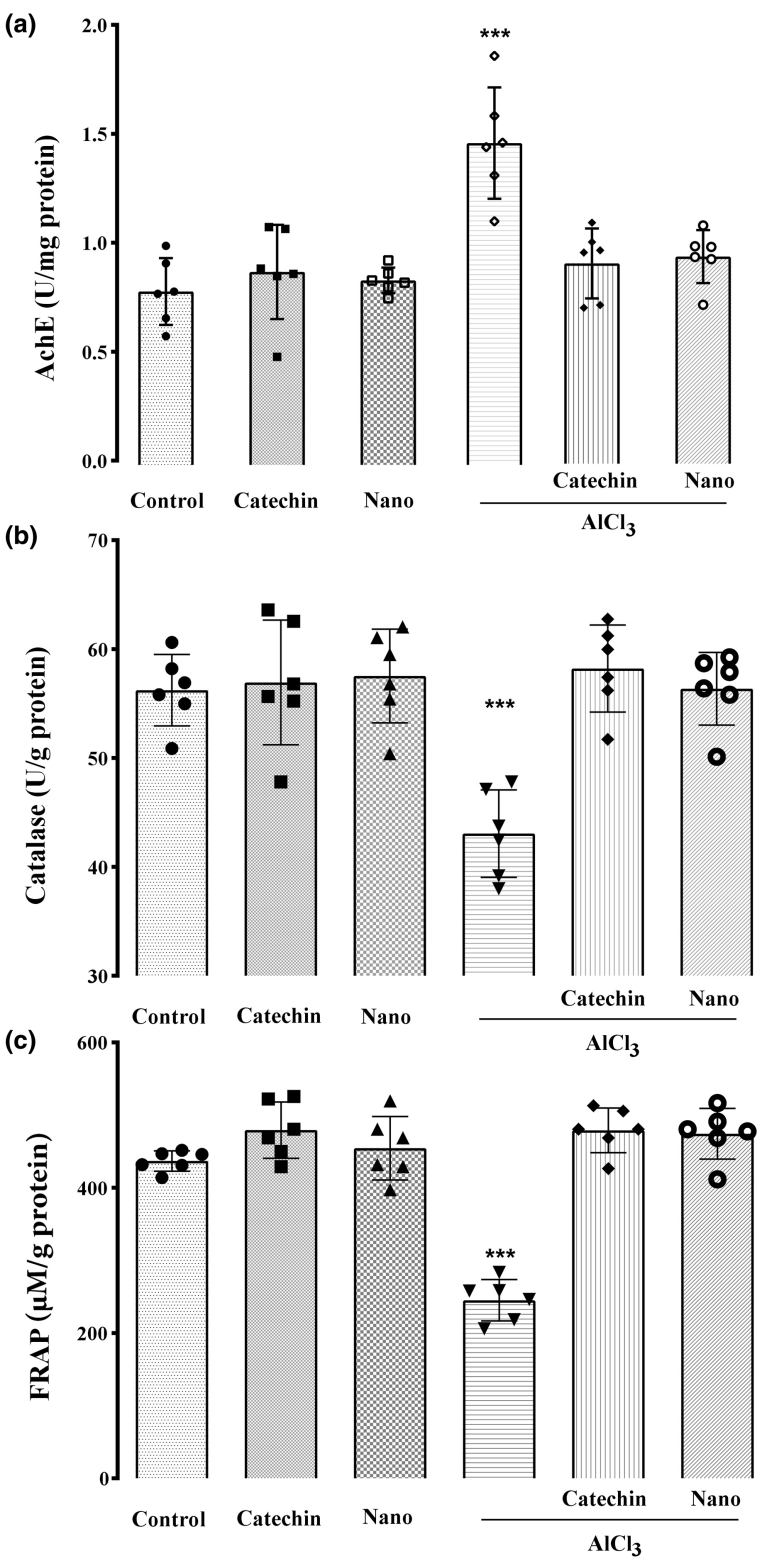
The results of the statistical analyses of the biochemical enzymes. (a) The amounts of AchE enzyme activity in the studied groups: Administration of AlCl_3_ has significantly increased the activity of AchE enzyme in hippocampus of rats group animals as compared to control. (b) Catalase enzyme activity of the studied groups: the enzyme activity of catalase showed a significant decrease in AlCl_3_ rat group compare to the control group, and (c) the amounts of total antioxidant according to FRAP value: There was a significant decrease in the amount of total antioxidants in the hippocampus of AlCl_3_ rat group animals as compared to control. values are expressed Mean ± SD and (***) indicate significant differences compared to the control group (*p* < 0.001).

As shown in Figure 6a, the administration of AlCl_3_ to rats induced significant increase in AChE activity in the hippocampus compared to the control group (1.45 vs. 0.78 U/mg protein) (*p* < 0.05) (Figure 6a). The administration of catechin and catechin‐loaded chitosan‐alginate NPs to rats improved AChE activity significantly (0.90 and 0.96 U/mg protein, respectively) when compared to the AlCl_3_ group (*p* < 0.05). However, no significant difference was observed in AChE activity in catechin and catechin‐loaded chitosan‐alginate NPs treated rats as compared to the control group (*p* > 0.05). In addition, no significant difference was found in AChE activity in the group of rats treated with catechin in comparison to the group treated with catechin‐loaded chitosan‐alginate NPs (*p* > 0.05) (Figure 6a).

### 3.4 Oxidative stress biomarkers in brain

The amount of the CAT enzyme activity in the control group, AlCl_3_ treated group, catechin group, nanocarrier group, treatment group 1 (AlCl_3_ + catechin), and treatment group 2 (AlCl_3_ + nanocarrier) were 56.23 ± 3.28, 43.05 ± 4.01, 56.93 ± 5.730, 57.53 ± 4.30, 56.36 ± 3.33 and 58.21 ± 3.99 U/g protein, respectively. Thus, these amounts for TAC in the control group, AlCl_3_ treated group, catechin group, nanocarrier group, treatment group 1 (AlCl_3_ + catechin), and treatment group 2 (AlCl_3_ + nanocarrier) were 436.87 ± 14.04, 245.11 ± 28.49, 479.30 ± 38.75, 454.40 ± 43.73, 479.02 ± 30.80 and 472.27 ± 34.90 U/mg protein, respectively. Rats administered AlCl_3_ revealed a significant decrease in CAT (43.05 vs. 56.23 U/g protein) and TAC (245.11 vs. 436.87 μM/g protein) of hippocampus as compared to the control group (*p* < 0.05). However, treatment of rats with catechin and catechin‐loaded chitosan‐alginate NPs was ameliorated the decrease in both markers; such that, a significant difference was found in CAT and TAC in the rats treated with catechin (56.36 U/g protein and 479.02 μM/g protein, respectively) and catechin‐loaded chitosan‐alginate NPs (58.21 U/g protein and 472.27 μM/g protein, respectively) when compared to the AlCl_3_ group (*p* < 0.05). However, there was no significant difference in CAT and TAC in the rats treated with both forms of catechin compared to the control group (*p* > 0.05). In addition, no significant difference was found in CAT and TAC in the group of rats treated with catechin in comparison to the group treated with catechin‐loaded chitosan‐alginate NPs (*p* > 0.05) (Figure 6b,c).

### 3.5 Spatial learning and memory

**FIGURE 7:**
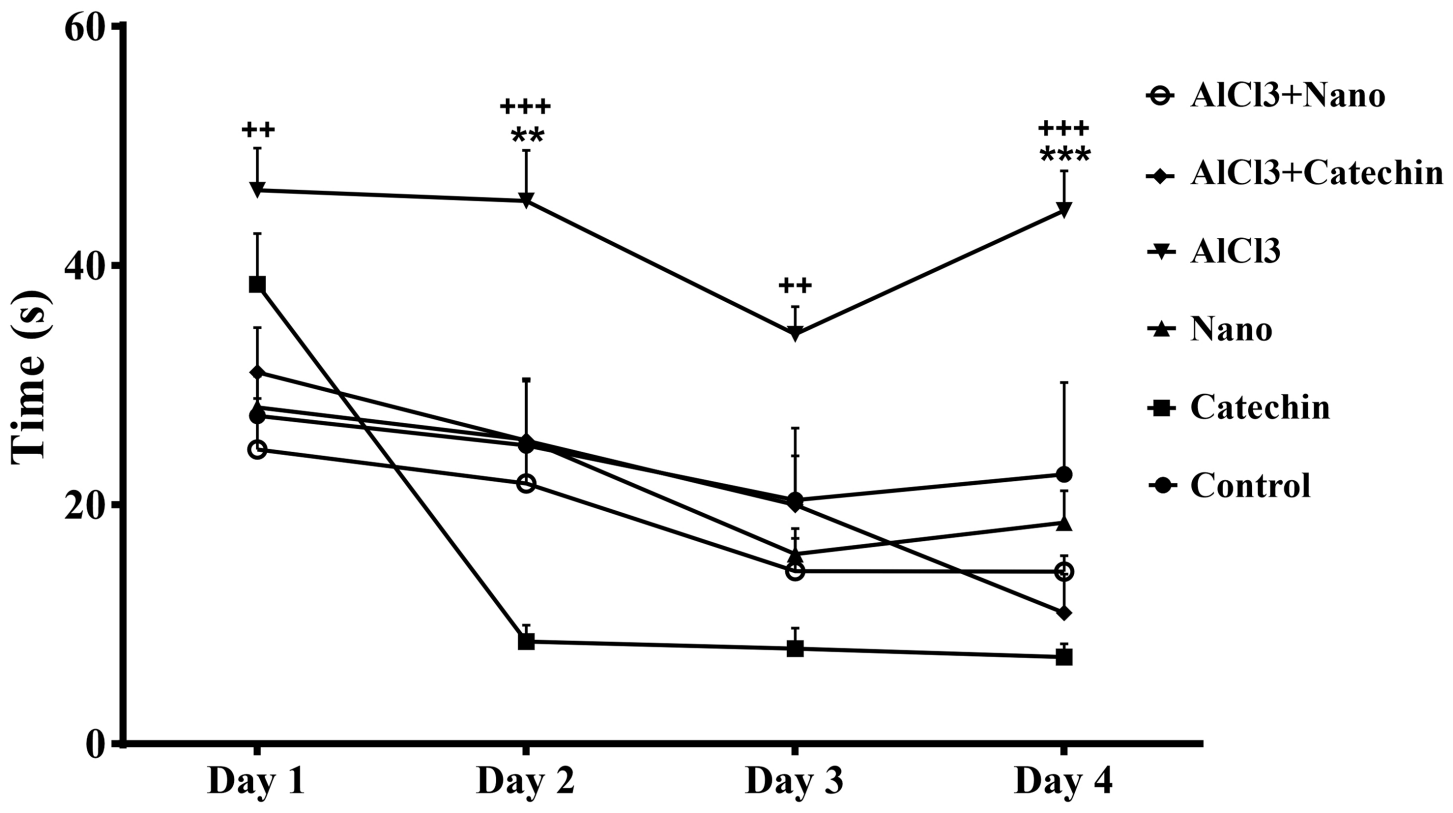
Morris water maze trail day's results. The results show that chitosan‐alginate nanoparticles containing drug catechin improve the cognitive function in four tail days in both treated groups in comparison to AlCl_3_ treated group. Each column shows Mean ± SEM and **p < 0.01, ***p < 0.001 significantly compare to control group and ++*p* < 0.01, +++*p* < 0.001 significantly different from AlCl_3_ group.

**FIGURE 8:**
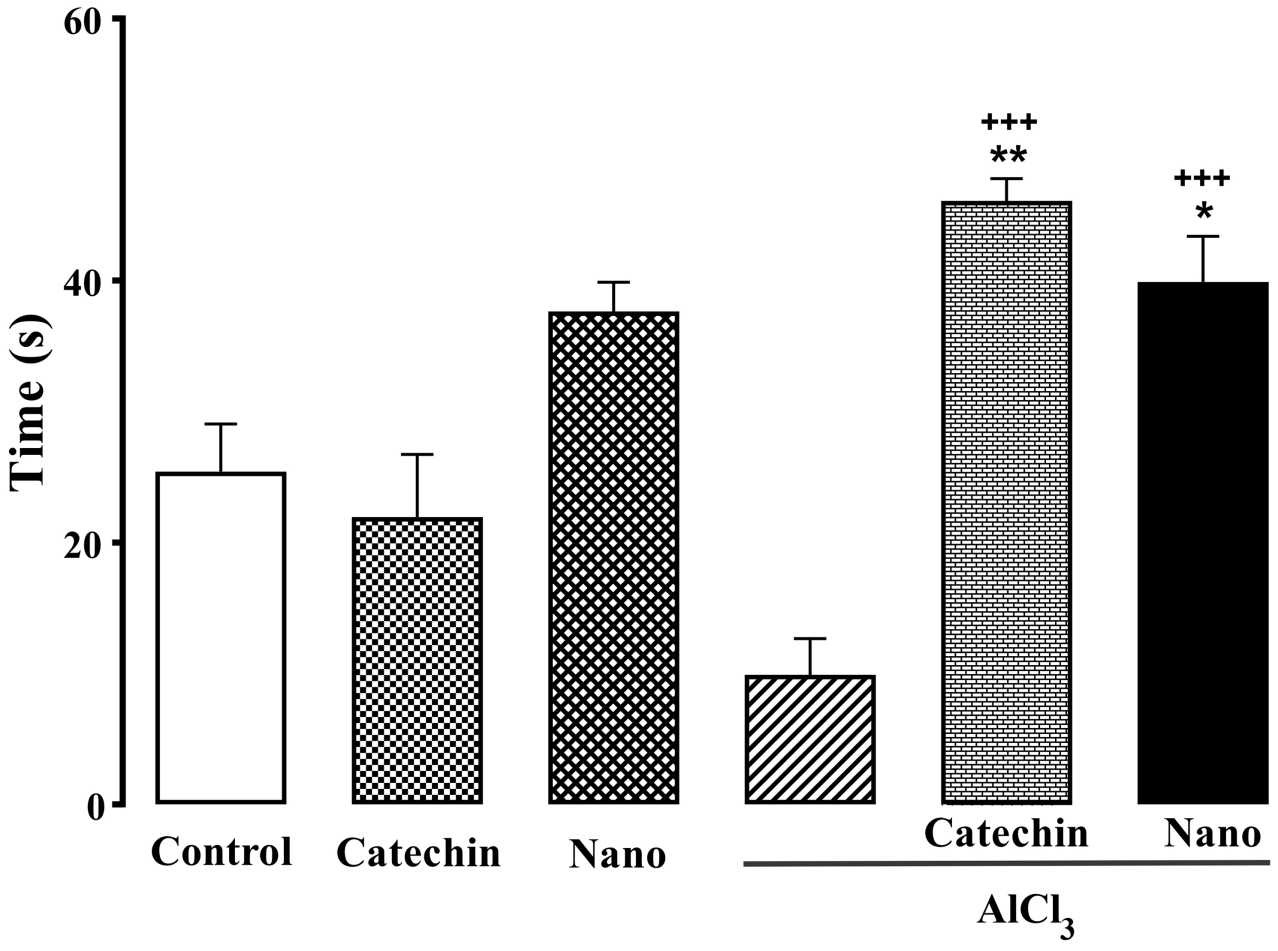
Morris water maze probe day's results. Probe day's results indicate that AlCl_3_ treated group significantly spent less time in target quadrant. However, that this time is more over in both catechin and nanocarrier‐catechin received groups. Each column shows Mean ± SEM and *p < 0.05, **p < 0.01 significantly compare to control group and +++*p* < 0.001 significantly different from AlCl_3_ group.

In the MWM test, rats administered AlCl_3_ significantly took more time to reach the platform in days one, two, and four compared to the control group during 4 days (*p* < 0.05). However, the rats treated with catechin‐loaded chitosan‐alginate NPs significantly spent less time before reaching the platform across four days in comparison to the AlCl_3_ group (*p* < 0.05) (Figure 7). As shown in Figure 8, AlCl_3_ treated rats spent significantly less time in the target quadrant compared to the control group (*p* < 0.05). However, the treatment of rats with catechin and catechin‐loaded chitosan‐alginate NPs significantly increased the time spent in the target quadrant than AlCl_3_ group (*p* < 0.05).

## 4 DISCUSSION

AD is a progressive neurodegenerative disorder affecting millions of individuals all around the world without any definitive treatment option (Association As, 2019). The exact mechanisms of AD are unclear; however, several factors such as oxidative stress and defects in cholinergic neuron transmission are known to be effective factors in the pathogenesis of this disease (Misrani et al., 2021; Terry & Buccafusco, 2003). The aim of the present study was to investigate the therapeutic effect of catechin loaded in chitosan‐alginate NPs and its comparison with free catechin in the AlCl_**3**_‐induced rat model of AD. In this study, catechin loaded in chitosan‐alginate NPs was prepared with 60% efficiency similar to the reports presented by Mandal et al. and Bhatt et al (Bhatt et al., 2019; Mandal et al., 2019). According to the results, NPs had a size distribution ranging from 40 to 45 nm, indicating that their smaller size may improve delivery to the intestinal epithelium. Furthermore, Chitosan‐Alginate NPs had a negative zeta potential (about −43.1 mv), suggesting stability and appropriateness for long‐term storage. Previous researches have shown a substantial association between zeta potential and the durability of NPs (Ing et al., 2012); the negative zeta potential seen in chitosan‐alginate NPs attributed to the carboxyl groups present in alginate (Dubey et al., 2016). On the other hand, the increased pH, which causes chitosan deprotonation, is related with bigger particle size and a decreased zeta potential (Ji et al., 2019). Consequently, pH seems to influence the swelling of chitosan. The results of the release test showed that NPs did not release in the acidic environment of the stomach, but their release increased in neutral settings such as blood circulation as the pH increased. The NP production size was strongly affected by Chitosan concentration based on other studies. There is no doubt that higher Chitosan concentrations produce larger NPs (Ali et al., 2011). As part of our study, we examined the initial concentration of Chitosan solution and discovered that NPs with a chitosan content of 0.1% had the optimum average size. These results highlight our carrier's encapsulating characteristics and imply that it could improve catechins' oral bioavailability.

Previous evidence has suggested that AlCl_3_ acts as a neurotoxin and can cause impaired cholinergic neurotransmission mainly by affecting the synthesis and release of neurotransmitters such as Ach which plays an essential role in memory formation and recovery in the central nervous system (Chiroma et al., 2018; Liaquat et al., 2019). Impaired cholinergic transmission is associated with the severity of dementia and neuropathological alterations in AD patients (Terry & Buccafusco, 2003). AlCl_3_ administration induces Ach scarcity because of decreased Ach release, and choline acetyltransferase (ChAT) activity as well as increased AChE activity (Singh et al., 2013). Additionally, oxidative stress results in reduced activity of cell surface AChE due to the alteration of membrane fluidity (Singla & Dhawan, 2013). In line with the results of previous studies, our findings showed that AlCl_3_ treatment significantly increased the activity of AChE in the brain (Borai et al., 2017; Haider et al., 2020; Mahdy et al., 2012). The results of the present study showed that both catechin and catechin‐loaded chitosan‐alginate NPs decreased the activity of AChE in AD rats, suggesting the effect of catechin on cholinergic neurotransmission. The reduction of AChE activity by the administered encapsulated form of catechin in chitosan‐alginate NPs containing lower amounts of catechin (10 mg/kg) compared to the free catechin (50 mg/kg) indicates the high efficacy of catechin‐loaded chitosan‐alginate NPs for the treatment of AD. Additionally, we assume that the use of chitosan‐alginate NPs coating can reduce catechin toxicity and its side effects compared to free catechin. Limited data are available regarding the side effect profile of free catechin such as hepatotoxicity (Additives EPoF, Food NSat, et al., 2018), restlessness, confusion, nausea, vomiting, and headaches (Jatoi et al., 2003). In the present study, the group of rats treated with free catechin showed higher rate and speed of movement, as indicatives of restlessness or confusion as related side effects of free catechin, than the group treated with catechin‐loaded chitosan‐alginate NPs. However, further studies are needed to confirm the hypothesis as well as the full potential of nanoformulated catechin as a therapeutic agent.

It has been demonstrated that oxidative stress plays an important role in the pathophysiology of AD. Free radicals such as reactive oxygen species (ROS) originating from metabolic processes or environmental pathogens, are contributed to the pathogenesis of AD through mitochondrial dysfunction and peroxidation of macromolecules (Cassidy et al., 2020; Misrani et al., 2021). AD‐induced rats showed a significant reduction in the levels of hippocampus CAT and TAC as compared to controls. This result confirms that AlCl_3_ **–** induced neurotoxicity is associated with oxidative stress. The result agrees with previous research showing altered oxidative stress due to an increment of ROS and reduced free radical scavenging in AlCl_3_ – induced neurotoxicity (Abu‐Taweel & Al‐Mutary, 2021; Mohamed et al., 2021; Prema et al., 2017). An increase in the level of free radicals causes damage to the mitochondrial membrane and disruption of the respiratory chain and finally cellular damage in the nervous system (Higgins et al., 2010). In the current study, rats treated with both catechin and catechin‐loaded chitosan‐alginate NPs showed significantly enhanced levels of CAT and TAC to normal levels; indicating that catechin can protect against cell oxidative injury as reported previously in in vitro and in vivo studies (Bernatoniene & Kopustinskiene, 2018). However, the treatment with catechin‐loaded chitosan‐alginate NPs was not superior in reducing oxidative stress to free catechin. This finding explains one of the potential mechanisms through which catechin improves cognitive activity and prevents AD in Wistar male rats.

In the present study MWM test was used to examine the spatial learning and memory of AD rats. Treatment of AD rats with catechin‐loaded chitosan‐alginate NPs showed a significantly reduced time before reaching the platform across 4 days compared to rats treated with AlCl_3_. Additionally, treated rats with catechin and catechin‐loaded chitosan‐alginate NPs markedly increased the time spent in the target quadrant than rats treated with AlCl_3_. This finding is in accordance with previous reports showing that catechin can improve memory impairment in various rat models of AD (Ahmed et al., 2013). The results of a study by Ahmed et al indicated that the administration of catechin hydrate in a rat model of AD induced by streptozotocin significantly ameliorates the impairment in memory and learning evaluated by the MWM test (Ahmed et al., 2013). In addition, another study by Rezai‐Zadeh et al showed that the administration of EGCG is beneficial for cognitive function in Alzheimer transgenic mice (Rezai‐Zadeh et al., 2008). However, to the best of our knowledge, no previous study has examined the therapeutic effect of catechin loaded in NPs on memory in AD rats.

## 5 CONCLUSION

The present study revealed that the administration of catechin‐loaded chitosan‐alginate NPs was efficacious in ameliorating aluminum‐induced neurotoxicity in AD rats through attenuation of AChE and oxidative stress. Furthermore, it improved the functional outcomes as represented in behavioral test. In general, this study indicated that the use of catechin‐loaded chitosan‐alginate NPs is a beneficial therapeutic option against behavioral and chemical alteration of AD.

## AUTHOR CONTRIBUTIONS

Fatemeh Tohidi main idea and designed the study, Elnaz Mohammadbaghban performed the experiments, data collection and the manuscript drafting, Ali Taravati and Hossein Khaleghzadeh‐Ahangar contributed in data interpretation and Hossein Najafzadehvarzi participated in drafting manuscript and animal studies. All authors revised and approved the final version of manuscript.

## FUNDING INFORMATION

This study supported by Babol University of Medical Sciences as a master thesis (grant number: 724132168).

## CONFLICT OF INTEREST STATEMENT

The authors have no relevant financial or non‐financial interests to disclose.

## ETHICS STATEMENT

This study was performed in line with the principles of the Declaration of National Institute of Health Guide for the Care and Use of Laboratory Animals (Council). Approval was granted by the Ethics Committee of Babol University of Medical Sciences, Babol, Iran (IR.MUBABOL.HRI.REC1398.012).

## Data Availability

The data that support this study's findings are available from the corresponding author upon reasonable request.
